# ‘Get Healthy!’ physical activity and healthy eating intervention for adults with intellectual disability: results from the feasibility pilot

**DOI:** 10.1186/s40814-023-01267-5

**Published:** 2023-03-22

**Authors:** Carmela Salomon, Jessica Bellamy, Elizabeth Evans, Renae Reid, Michelle Hsu, Scott Teasdale, Julian Trollor

**Affiliations:** 1 Department of Developmental Disability Neuropsychiatry, UNSW Sydney, Room 241, Level 2, Biolink Building E25, Sydney, NSW 2052 Australia; 2 School of Medical, Indigenous & Health Sciences, Faculty of Science, Medicine & Health, University of Wollongong, Wollongong, 2522 Australia; 3 Council for Intellectual Disability, 418A Elizabeth St, Surry Hills, NSW 2010 Australia; 4 The Boden Institute of Obesity, Nutrition, Exercise & Eating Disorders, The University of Sydney, Sydney, 2006 Australia; 5 Keeping the Body in Mind Program, South Eastern Sydney Local Health District, 26 Llandaff Street Bondi Junction, Sydney, 2022 Australia; 6 Discipline of Psychiatry and Mental Health, UNSW Sydney, Kensington, Australia

**Keywords:** Intellectual disability, Aging, Physical activity, Exercise, Nutrition, Protocol, Photographic food record, Accelerometry, Intervention, Health promotion

## Abstract

**Background:**

People with intellectual disabilities (ID) experience high rates of lifestyle related morbidities, in part due to lack of access to tailored health promotion programmes. This study aimed to assess the feasibility and preliminary efficacy of a tailored healthy lifestyle intervention, *Get Healthy!*

**Methods:**

*Get Healthy!* is a 12-week physical activity and healthy eating programme designed to address lifestyle-related risks for adults with mild-moderate ID. The feasibility pilot was designed to assess subjective participant experience and programme feasibility across: recruitment and screening, retention, session attendance and engagement, adverse events, and practicality and reliability of outcome procedures. Exploratory programme efficacy was assessed across the following measures: anthropometry (body mass index, weight, waist circumference), cardiovascular fitness, physical strength, dietary intake, healthy literacy, and quality of life.

**Results:**

Six participants with moderate ID and two carer participants completed the feasibility trial, representing a 100% retention rate. Qualitative data indicated the programme was well received. Participants with ID attended 75% of sessions offered and displayed a high level of engagement in sessions attended (91% mean engagement score). While most data collection procedures were feasible to implement, several measures were either not feasible for our participants, or required a higher level of support to implement than was provided in the existing trial protocol. Participants with ID displayed decreases in mean waist circumference between baseline and endpoint (95% CI: − 3.20, − 0.17 cm) and some improvements in measures of cardiovascular fitness and physical strength. No changes in weight, body mass index, or objectively measured knowledge of nutrition and exercise or quality of life were detected from baseline to programme endpoint. Dietary intake results were mixed.

**Discussion:**

The *Get Healthy!* programme was feasible to implement and well received by participants with moderate ID and their carers. Exploratory efficacy data indicates the programme has potential to positively impact important cardiometabolic risk factors such as waist circumference, cardiovascular fitness, and physical strength. Several of the proposed data collection instruments will require modification or replacement prior to use in a sufficiently powered efficacy trial.

**Trial registration:**

ACTRN: ACTRN12618000349246. Registered March 8th 2018—retrospectively registered, https://www.anzctr.org.au/Trial/Registration/TrialReview.aspx?id=374497 UTN: U1111-1209–3132.

## Key messages regarding feasibility

- There was uncertainty regarding the feasibility of implementing the *Get Healthy!* group programme for adults with mild-moderate ID, and whether the selected outcome measures could be reliably administered to the population.
- The *Get Healthy!* programme was feasible to implement, however, several outcome measures required a greater level of training/support to administer than was provided in the feasibility protocol, and a small number were too complex for the participants with moderate ID.
- The *Get Healthy!* programme will be feasible to administer in a sufficiently powered trial; however, several screening and outcome measures will require modification prior to trial commencement.

## Background

Despite significant advances in longevity and quality of life, people with intellectual disabilities (ID) continue to experience poorer health outcomes than the general population [1]. The term ‘intellectual disability’ is used to describe any person who experiences ‘significant limitations both in intellectual functioning and in adaptive behaviours, as expressed in conceptual, social and practical adaptive skills. The disability presents or originated during the developmental period before the age of 18 years’ [2]. Many causes of premature mortality in this population are linked to potentially preventable conditions [3]. Lifestyle risks including poor diet quality [4], low levels of physical activity [5], and high rates of sedentary behaviour [6], are prevalent across age groups. People with ID are more likely than the general population to be overweight or obese and have high rates of type 2 diabetes and lipid abnormalities [7, 8]. Common prescribing of high cardiometabolic liability psychotropics in this population [9] further exacerbates risk. Health status, quality of life and health expenditure are all negatively impacted by this high prevalence of lifestyle-related diseases [10, 11].

Tackling lifestyle-related behaviour has been identified as a priority area for improving health outcomes for people with ID [12]. However, people with ID still have low levels of engagement in health promotion initiatives and preventative screenings [1]. Financial, physical, social and disability related barriers limit this population’s ability to access health promotion programmes available to the general population [13]. The limited and inconsistent ID health training received by the medical and allied health workforce [14, 15] means that many care providers lack confidence tailoring health promotion practices to the unique needs of this group.

There is also a lack of clarity regarding the essential components of lifestyle change interventions most likely to improve health outcomes. Evidence for the efficacy of general population healthy lifestyle programmes is robust [16]; however, these programmes are not necessarily generalisable to people with ID. Results from ID population-specific interventions reported in the literature are limited and have had mixed results. Weight loss for adults with ID, for example, has been inconsistently reported across interventions, but appears to be most likely in the context of multi-modal interventions encompassing physical activity, dietary and behaviour change components—see [17] for a review. Methodological weaknesses, use of varied outcome measures and differing population characteristics (i.e. level and cause of ID, age-group, gender, living arrangements) across studies limits comparison of findings [18]. A meta-analysis of randomised-controlled healthy lifestyle trials for adults with ID showed statistically significant improvements in waist circumference only [18].

Further trials are needed to clarify the core components of interventions that will promote engagement and positive lifestyle change in this population. The primary aim of this study is to assess the feasibility of implementing a tailored healthy lifestyle programme, *Get Healthy!* with adults with mild-moderate ID. The secondary study aim is to explore potential programme efficacy. Results from the feasibility pilot will be used to refine the programme content and data collection protocol prior to undertaking a sufficiently powered efficacy trial.

### Background to the ‘Get Healthy!’ programme

*Get Healthy!* is a 12-week multi-modal lifestyle intervention programme focusing on physical activity and healthy eating for adults (40 + years) with mild to moderate ID, however is suitable for all adults with ID. The programme was developed by a consortium of topic experts in the fields of nutrition, ID, ageing, exercise physiology, nursing, psychiatry, and psychology. A series of focus groups with adults with ID and their paid carers [19] contributed consumer input to the programme design. Table 1 summarises the setting, structure and content of the programme, and lists all behaviour-change techniques used in the programme delivery.

**Table 1:** ‘GET HEALTHY!’ Summary of program structure and content

**Program setting:** Lifestyle Clinic in tertiary research institute		
**Program structure:** 12-week small group program consisting of three face-to-face contact hours per week (1x one hour nutrition session per week plus 2x one hour physical activity sessions held on non-consecutive days). Physical activity sessions were delivered by a practicing Accredited Exercise Physiologist. Nutrition sessions were delivered by Accredited Practicing Dietitians. All instructors had previous experience working with people with intellectual disability		
	**Program Content**	
	*Physical Activity*	*Healthy Eating*
	**10% didactic information** Topics covered: What it means to be healthy; Consequences of obesity; Physical activity and screen time guidelines; Appropriate goal setting; Planning for maintenance and self-management; Barriers to Physical activity and how to address them **40% aerobic exercise** **30% strength-based exercise** **20% balance-based exercise**	**90% didactic content** Topics covered: The five food groups; Discretionary foods and healthy snacks; Healthy drinks; Portion size and mindful eating; Eating out choices **10% practical food related outings/preparation**
**CALO-RE Behavior-change techniques used**^a^		
**1. Information provision (general)**	✓	✓
**2. Information provision (to the individual)**	✓	✓
**3. Information provision (others’ approval)**		✓
**4. Information provision (others’ behavior)**	✓	✓
**5. Goal setting (behavior)**	✓	✓
**6. Goal Setting (outcome)**	✓	
**7. Action Planning**	✓	✓
**8. Identifying barriers/Problem resolution**		✓
**9. Setting graded tasks**	✓	✓
**10. Review of behavioral goals**		✓
**11. Review of outcome goals**	✓	✓
**12. Effort or progress contingent rewards**	✓	✓
**13. Successful behavior contingent rewards**	✓	✓
**14. Shaping**		
**15. Generalization of target behavior**		
**16. Self-monitoring of behaviour**		
**17. Self-monitoring of behavioral outcome**		
**18. Focus on past success**	✓	✓
**19. Provide feedback on performance**	✓	✓
**20. Informing when and where to perform the behavior**	✓	✓
**21. Instruction on how to perform the behavior**	✓	✓
**22. Demonstrate behavior**	✓	✓
**23. Training to use prompts**		
**24. Environmental restructuring**		✓
**25. Agreement of behavioral contract**	✓	✓
**26. Prompt practice**	✓	
**27. Use of follow-up prompts**		
**28. Facilitate social comparison**	✓	
**29. Plan social support**		
**30. Prompt identification as role model**	✓	✓
**31. Prompt anticipated regret**		
**32. Fear arousal**	✓	
**33. Prompt self talk**	✓	
**34. Prompt use of imagery**	✓	
**35. Relapse prevention**		
**36. Stress management**		
**37. Motivational interviewing**	✓	
**38. Time management**		
**39. Communication skills training**		✓
**40. Stimulate anticipation of future rewards**	✓	

^a^*CALO-RE* Taxonomy of Behavior Change. (PDF Download Available). Available from: https://www.researchgate.net/publication/274512164_CALO-RE_Taxonomy_of_Behavior_Change_Techniques [accessed Mar 29 2018]

## Methods

The full feasibility pilot protocol has been published elsewhere [20]. Methodology is summarised below for convenience.

### Recruitment

Participants were recruited through disability service providers proximal to the healthy lifestyle centre where the intervention was delivered in metropolitan NSW, Australia. Adults who were identified by carers/disability organisations as having mild to moderate ID and concerns about cardiometabolic health were eligible to participate in the programme. The participants’ main carers were also invited to participate either independently in the full programme (carer-protocol A) or as a support person to the enrolled participants with ID (carer-protocol B). Participants who were non-ambulatory, had severe-profound ID, or who were not cleared by their general practitioner (GP) to participate due to either high physical or psychiatric risk, were excluded.

### Consent

Written informed consent was obtained from all participants prior to trial commencement. For participants who lacked capacity to consent (~ 70%), written consent was provided by their legal guardian/carer as required by law. All participants with ID also obtained a signed medical clearance from their GP prior to enrollment. The study was conducted in accordance with the ethics approval granted by the UNSW HREC (Approval number: HC17471).

### Data analysis

Programme feasibility was assessed across the domains of recruitment and screening, retention, adverse events, session attendance and session engagement. Every session the programme facilitators recorded attendance and scored attendees based on their level of engagement in the session (0 = did not attend, 1 = participated minimally, 2 = participated moderately well to very well). At the completion of the intervention combined scores for every session attended were used to categorise participants into high (75–100%), medium (50–74%) or low engagement (< 50%) groups. Subjective participant experience was gathered in audio-recorded semi-structured exit interviews with all participants. Qualitative data from exit interviews was transcribed and thematically organised using the software programme NVivo (version 11.0.0).

All outcome measures included in the trial are listed in Table 2. The Statistical Package for Social Science (SPSS) was used to analyse percentages, score means and/or frequencies where relevant. Acknowledging the small sample, we used 95% confidence intervals to reported outcomes in order to provide a clinically relevant indication of the direction of the effect being measured.

**Table 2:** Clinical outcome measurements/procedures used in the *‘Get Healthy!’* feasibility trial

Dimension measured	Procedure details
Body mass index (BMI)	BMI = weight/height (kg/m^2^) [22]
Waist circumference	Measured at the midpoint between the iliac crest and the lowest rib, in full expiration, to the nearest 0.1 cm while the person is standing [23]
Blood pressure	To be measured using sphygmomanometer while the participant is seated and has rested for at least 5 min prior [24]
Cardiovascular fitness	YMCA sub-maximal ergometer test 12 min duration [25]: - Minutes/stages performed - Peak heart rate (%APMHR) - Peak workload achieved
Physical activity level and sedentary behaviour	*Subjective data:* - International Physical Activity Questionnaire-proxy respondent (IPAQ-pr) proxy report [26] *Objective data:* - Waist-based GTX3 actigraph accelerometer to be worn for a period of 3–5 days in each data collection period [27]
Physical Strength	- 30-s modified push-up test [28] - Medicine ball throw/chest pass [29] - 10 RM testing [30] - 30-s sit-to-stand test [31]
Quality of life	Personal Wellbeing Index-Intellectual Disability (PWI-ID) [32]
Dietary intake	- 3-day photographic food record [33] - Proxy-assisted 24-h recall [34]
Healthy literacy	Nutrition and Activity Knowledge Scale for Use with People with an Intellectual Disability (NAKS) questionnaire [35]

Food intake data was calculated from photographic food and drink records at baseline and endpoint. Data was interpreted and analysed by two Accredited Practicing Dietitians using Foodworks® (version 9) nutrition analysis software (Xyris Software, 2018). Days with less than three meals captured were removed prior to analysis. A Healthy Eating Index for Australian Adults (HEIFA) [21] was then applied to determine overall diet quality.

## Results

Six participants with ID and two carer participants completed the full screening process and were enrolled in the trial.

### Participant demographics

Table 3 summarises demographics of participants with ID.

**Table 3:** Demographics of participants with ID

Age (years)	Gender	Level of ID	Mobility status	Type of residence	Co-morbidities
Mean: 46 SD: 13 Range: 28–62 ^a^	Male (*n* = 4) Female (*n* = 2)	Moderate ^b^ (*n* = 6)	Able to ambulate independently (*n* = 5) Ambulate with cane (*n* = 1)	Group disability housing (*n* = 4) With family (*n* = 1) Independently (*n* = 1)	Obese (*n* = 3) Overweight (*n* = 2) Autism (*n* = 1) Impaired glucose tolerance (*n* = 1) Ventricular septal defect, and valvular heart anomalies (*n* = 1)

^**a**^Four of the six participants were aged 40 years and over. A further two participants below this age bracket were included because they expressed an interest in improving cardiometabolic fitness

^b^Level of ID was based on the assessment of the research team delivering the ‘Get Healthy!’ intervention

### Carer participant demographics

Due to competing time commitments and variations in work schedules no family members or paid carers were able to enrol in the full programme (carer-option A). Two paid carers enrolled in the option B participation pathway. This participation pathway involved attending sessions in a support capacity as able. Both enrolled carer participants were female, over 18 years of age, and employed as paid disability staff. They supported several of the participants with ID in residential and day care settings. On average, these carers attended approximately 50% of the available sessions. Since carer protocol B did not include collection of outcome measures data, all efficacy data reported below pertains to the participants with ID only Table 4.

### Feasibility outcomes

#### Recruitment and screening

Recruitment was completed between July 2017 and February 2018. Thirty people with ID expressed an initial interest in participating in the trial programme. Of these, 14 were either unable to complete the consent form, or unable to determine a suitable time to attend the initial assessment. Sixteen participants completed the consent form and participated in the initial assessment. Ten participants dropped out during this screening process. Reasons for drop-out during the screening process included:

- Scheduling and/or transport problems (*n* = 7)
- Having a level of ID (severe to profound) that meant the person was unable to participate in the group learning structure of the ‘Get Healthy!’ programme (*n* = 3).

**Fig. 1:**
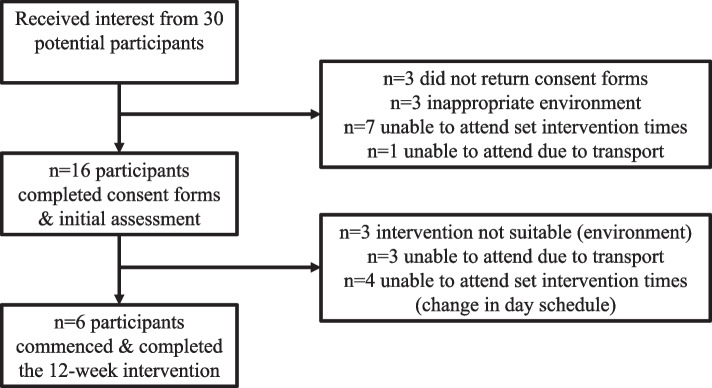
Consort diagram of participant recruitment

GPs screened each of the remaining six participants and provided signed consent for their participation in the feasibility trial (Fig. 1). Recruitment was ceased in February 2018 (6 months) in accordance with the funding allocated.

#### Retention rate

All six participants with ID who completed the full screening process and enrolled in the study went on to complete the programme, representing a 100% retention rate.

#### Attendance and session engagement

On average, participants with ID attended 75% of sessions offered as part of the programme. Attendance rates at physical activity and nutrition sessions were similar (74% and 76% respectively). The top reason participants missed scheduled sessions was to attend medical, allied health or dental appointments that had been arranged prior to study enrolment. Mean participant engagement scores across all sessions attended was ‘high’ (91%); however, participants were significantly more engaged in the physical activity sessions compared to the nutrition sessions (respective mean engagement scores of 99% versus 77%).

#### Outcome measure feasibility

Table 4 summarises the feasibility of all outcome measures according to whether they were.

(i) Reliably administered to all participants at both baseline and endpoint, or.
(ii) Either unable to be administered *or* administered but returned incomplete or unreliable data sets.

For all outcome measures where problems with data reliability or completeness were noted, specific issues of concern are listed. No adverse events were experienced by any participants.

**Table 4:** Feasibility of outcome measures

**OUTCOME** **MEASURE**	**Measure completed reliably and fully at both time points**	**Incomplete data returned or problems with measure validity noted**	**Identified problems with administration and or validity of measure**
Anthropometric measures	X		
Measures of cardiovascular fitness	X		
Measures of physical strength (excluding 10RM)	X		
Knowledge Scale for Use with People with an Intellectual Disability (NAKS) questionnaire		X	While all participants completed this measure, wide variability in baseline to endpoint scores raise questions about instrument reliability for our cohort: For example, one participant scored 13 at baseline but went on to score a significantly lower score of 5 at endpoint. Since it is unlikely that participants would ‘lose’ this amount of knowledge in a 12-week time frame it is possible that scores reflect guess-work rather than change in knowledge
The Personal Wellbeing Index- Intellectual Disability (PWI-ID)		X	While all participants agreed to undertake the measure significant differences in pre-testing scores from baseline to endpoint raise concerns about instrument validity in our cohort: At baseline two participants were unable to complete step two of the pre-testing process. We were therefore unable to administer the measure to them. However, at endpoint, the same two participants were able to complete the full pre-testing protocol and the 11-point scale. The extremely high scores these participants recorded on the measure at endpoint (100 and 92.9 respectively) raise questions about the reliability of their responses, however. At baseline the remaining four participants pre-testing scores indicated that they were unable to complete the 11-point scale, however, at endpoint they were all able to appropriately answer the pre-testing questions and thus had the 11-point scale administered to them
24 hour food recall		X	While this form was handed to each participant and the support worker who attended the session with them, no completed or partially completed forms were returned at baseline or endpoint: Participants were unable to independently recall what they had eaten at previous meals, and family members and carers did not complete the form on their behalf
Food photography		X	Only two participants provided photographic data at both the pre-and post-program data collection periods. While the two participants captured three full days at baseline, neither reached the target of a three complete photographic records at endpoint (capturing 1 and 2 days only). One participant declined to undertake this task at both time-points (reason was not stated). The remaining three participants either did not take photos despite agreeing to undertake the task, took incomplete days of records or took photos in which they had blocked the camera lens with their hand or clothes
Accelerometer data		X	One participant (baseline) and two participants (endpoint) did not meet the minimum wear time of at least six hours on three out of the five wear days that was stipulated in our protocol. One participant who had a co-occurring diagnosis of autism, struggled with wearing the device due to sensory issues (stated he dislikes the feel of the device around his waist)
IPAQ-proxy		X	While this form was handed to each participant and the support worker who attended the session with them, only two of the forms were returned at baseline or endpoint, and these were insufficiently completed to provide meaningful data
Physical Strength: 10RM strength testing		X	We were unable to reliably establish participants’ rate of perceived exertion in the pre-testing phase of the protocol and thus were unable to administer this outcome measure. Inability to establish perceived rate of exertion was related to difficulties participants experienced using even a modified scale to rate their level of exertion. For example, participants, both in cases where the weight used was extremely light and in cases where the weight used was so heavy the participants could not attempt the task, reported the exercise as “easy”. Without this baseline measurement, all participants commenced the program on the lowest weight available and the decision to increase weight was based on technique alone and experienced Exercise Physiologist decision making

### Clinical outcomes

#### Anthropometric measures

Table 5 lists the groups’ mean baseline and endpoint anthropometric data. There was a decrease in the groups mean waist circumference (WC) from baseline to endpoint (95% CI: − 3.20, − 0.17 cm). Individually, one participant gained 0.5 cm in WC during the intervention, while all five other participants displayed reductions in WC (− 0.4 cm; − 2.2 cm; − 2.4 cm; − 3.4 cm; − 2.2 cm). There was no clinically significant change in the groups mean weight (95% CI: − 1.6, 1.9) or BMI (95% CI: − 0.80, 0.90) from intervention baseline to endpoint, with three participants displaying a non-significant increase in BMI post intervention, and three participants displaying a non-significant decrease.

**Table 5:** Anthropometric means (± SD) at intervention baseline and endpoint

	*N*	Baseline mean (± SD)	Endpoint mean (± SD)
Weight (kg)	6	79.87 (11.78)	80.00 (10.27)
BMI (kg/m^2)^	6	32.86 (7.53)	32.88 (7.12)
Waist Circumference (cm)	6	108.32 (16.02)	106.63 (15.50)

#### Cardiovascular fitness (CV fitness)

Table 6 lists the groups mean CV data at baseline and endpoint. The mean number of minutes participants were able to undertake the activity increased from baseline to endpoint (95% CI: 2.44, 7.73). Similarly, the mean number of stages participants were able to perform increased from baseline to endpoint (95% CI: 1.16, 3.24 stages). The peak workload participants were able to achieve also increased from baseline to endpoint (95% CI: 49.17, 64.98). While none of the six participants were able to complete the full protocol at baseline, three participants were able to complete the protocol at endpoint. There were numerical improvements for mean YMCA Peak HR and APMHR from baseline to endpoint.

**Table 6:** Cardiovascular fitness-means (± SD) at intervention baseline and endpoint

	Baseline mean (± SD) *N* = 6	Endpoint mean (± SD) *N* = 5
YMCA minutes completed	2.81 (2.19)	7.80 (1.64)
YMCA stages completed	.50 (.84)	2.6 (.55)
YMCA peak heart rate	125.83 (25.93)	129.60 (22.53)
YMCA % APMHR	71.47 (13.30)	74.74 (13.68)
YMCA peak workload	70.83 (43.06)	120.00 (57.00)

#### Physical strength

All physical strength parameters showed numerical improvements across the intervention The mean improvement in the Sit To Stand (STS) exercise of 2.93 (95% CI: − 0.18, 7.00) from baseline to endpoint is promising, given that an improvement of STS =  > 2 reps may be clinically significant [36] particularly in relation to falls risk Table 7.

**Table 7:** Physical strength-means (± SD) at intervention baseline and endpoint

	*N*	Baseline mean (± SD)	*N*	Endpoint mean (± SD)
30sec push-ups	5	16.20 (3.56)	6	16.83 (2.48)
5kg medicine ball chest pass (m)	6	2.49 (0.43)	6	2.74 (0.48)
30sec sit to stand	5	12.40 (2.88)	6	15.33 (2.73)

#### Structured aerobic exercise conducted throughout intervention

Cycling duration—session 1 started at 9.0 (± 2.0) min (*n* = 4) increased to 13.4 (± 1.4) min by session 24 (48.9% increase) (*n* = 6). Similarly, this is reflected by the distance cycled during each session—session 1 started at 2.7(± 1.3) km (*n* = 4), which increased to 5.6 (± 1.1) km by session 24 (107.4% increase) (*n* = 6).

#### Accelerometer data

Five participants (baseline) and four participants (endpoint) had sufficient accelerometer wear-time to meet the threshold for data analysis set in our protocol. Their results are summarised in Table 8 and Fig. 1.

**Table 8:** Objective physical activity data—means (± SD) at intervention baseline and endpoint

	Baseline mean (± SD) *N* = 5	Endpoint mean (± SD) *N* = 4
Sedentary	643.94 ± 198.07	652.74 ± 128.57
Light	108.02 ± 78.72	73.42 ± 27.08
Moderate	24.96 ± 13.38	29.73 ± 10.21
Vigorous	0.60 ± 0.97	0.36 ± 0.25
MVPA	25.56 ± 12.98	20.06 ± 17.42

Total MVPA across the week (pre) —105.35 ± 47.11 min (*n* = 5) vs. post-intervention 133.48 ± 73.21 min (*n* = 4).Those meeting the PA guidelines (150 min of moderate PA) pre: 1, increased to 2 post-intervention.

#### Food intake

Only two participants (E and A) completed the food photography task to a sufficient extent to allow for a preliminary analysis to be undertaken. Key nutrition baseline and endpoint data for these participants are summarised in Table 9. Both participants decreased their total fat and saturated fat intake from baseline to endpoint. Wholegrain intake improved at endpoint; however, results for refined grain intake were mixed. While participant E’s HEIFA score increased from baseline to endpoint, indicating better overall diet quality, Participant A’s score decreased over the same period. Overall, average daily energy was lower for both participants at endpoint, along with most macronutrient and many micronutrients. It is unclear if these patterns reflect real changes in diet quality or the fact that both participants recorded fewer complete days of data at endpoint compared with baseline.

**Table 9:** Food intake at intervention baseline and endpoint

Participant ID	A		E	
Timepoint	Baseline	Endpoint	Baseline	Endpoint
HEIFA score^a^	43	38	43.75	47
Whole grain	1.86928	2.588864	3.010851	3.972731
Refined grain	2.805117	2.017218	5.401268	5.543118
Energy DF (kj)	6973.163	5663.742	8558.675	4794.232
Protein(g)	91.17966	72.96273	92.15803	44.85995
Carbs available (g)	180.365	134.8632	245.0325	191.0486
Total fat (g)	57.93966	51.95127	72.11869	17.68252
Saturated fat (g)	20.43112	19.24836	30.19545	7.440686
Dietary fibre (g)	27.31206	22.57688	20.64223	19.43015

^a^Heifa score is out of 100 possible points- higher scores indicate better diet quality and correlates to greater adherence to national dietary guidelines

#### Health literacy

Results from this trial showed no difference in mean NAKS scores from baseline (15.17) to Endpoint (13.17). Two of the participants recorded higher scores at endpoint while the remaining three participants recorded lower scores at endpoint. As identified in Table 3, there were concerns about the reliability of these data. All six participants completed the NAKS questionnaire pre- and post-intervention (*n* = 6).

#### Quality of life

Only three of the six participants passed the baseline pre-testing phase for the PWI-ID measure. Matched pre-post intervention data for these three participants shows no significant change in mean quality of life scores (baseline mean 88.2 vs. endpoint mean of 83.3). One of the three participants showed an increased score at endpoint, while the other two recorded decreased scores. As identified in Table 3, there were concerns about the reliability of these data.

#### Participant experience

All participants with ID, along with the two carer participants, participated in exit interviews. Qualitative feedback, including programme highlights and suggestions for improvement, were elicited, and thematically analysed. Core themes emerging from the exit interviews are summarised below.

#### Programme benefits

Participants highlighted several beneficial impacts from being involved in the *Get Healthy!* programme, including a sense of pride and achievement; improved knowledge of and commitment to healthy lifestyle change; increased opportunities for positive social interactions; and improved ability to set future healthy lifestyle goals. Table 10 provides contextualised data illustrating these positive impacts.

**Table 10:** Programme highlights and benefits: qualitative participant feedback

Programme highlight		Quotes from participants with intellectual disability	Quotes from carer participants
Programme fostered a sense of pride and achievement		“When I was riding the bikes [the instructor] would say, ‘come on, keep on going, keep on going’. I thought that was good because it made me feel like I was losing weight...pushing that bit more.” Participant F “I liked them [reward stickers] …That means I went on the bike with a high score… [so I was motivated] I did more”. Participant E	“I just really really like the whole [program] environment. I mean, [the instructor] was just amazing with the guys. She was always extremely positive. All the guys wanted to go each there because of her. … she was so calm and confident, that she was able to transmit that energy to the guys and then when they were there they wanted to be part of the activity, they wanted to be part of the exercise.” Carer 2 ““[Their] confidence grew with the [bike] equipment. In the beginning they were really hesitant and just did two minutes, then towards the end they were competing against each other- you know, pushing buttons themselves, and setting things up and all that sort of stuff. So the equipment confidence grew, they jumped in.” Carer 2
Programme Increased knowledge of and commitment to healthy lifestyles:	Healthy eating	“I liked learning about how much sugar was in the coca cola… “[I eat] better now [post-program]- at home, eat salad” Participant D “[I liked] learning about different foods, and what you can have and can’t have. I thought it [the program] was very good because it helped me out a lot.” Participant F “Well I [still] love eating food and sometimes I love ice-cream. But only one. Not every day”. Participant A “I eat a small amount now…a small one [plate of pasta] and vegetables and that…I started on the program, drinking it [water]…[and now I] eat fruit. Its really healthy- eat bananas and that and eat grapes” Participant E	“The guys used to come back and talk a lot about the healthy eating choices, and what they spoke about in the education sessions. And I know that they liked going out to different restaurants or shopping centres and participating in a healthy choice option. And there were a few of them that, you know, on days where they had brought their own morning tea or lunch in from home, you could see that they had made that healthy, conscious choice about buying the right things.” Carer 2
	Physical activity	“[I exercise more now because] It’s healthy for your heart” Participant D Yeah, walk- park- everyday [now]” …Exercise is very good. Very good idea. Makes sense. I loved it so much”. Participant A “I think exercising does you good because if you’re not exercising then you’re watching TV and I think you’ve got to get out, get away from it and go for a walk” Participant F	“A few of the guys [in the program], their doctors had actually reported improvements and were asking what was going on. One participant who was in the group, his respiratory doctor, said his lung capacity was, he couldn’t believe the change that had happened. Because you know that test where you blow into the thing and the balls go up- he said it was like a different person from when he did it the last time, to when he happened to do it just towards the end of the program.” Carer 2
The programme provided valued opportunities for social interaction		“[I liked exercising] with my friends… I liked the people that taught me how to do it [exercise] Participant F” “[I liked spending time with] the staff’ Participant E	“I think they enjoyed mixing with the university staff and the other different physiotherapists … they developed a bit of a friendship with a few people there. And also some of the other, some of the others participants- patients- some of the other older people coming in, they enjoyed having a chat with them every week as well” Carer 1 “They all had fun [doing the program together], they were always- because I used to drive them [to the program] on the Tuesdays- they were lined up at my car ready to jump in to go every week. I was never having to chase anyone or find out where they were.” Carer 2
The programme helped participants to identify future healthy lifestyle goals		“I do need help, because sometimes I’ve got problems with what I eat…I think I have to cut back on sweets. I eat too much from the top [of the food pyramid]…. [I’m going to] give up sugar, it’s not good. Too many Coca Colas not good” Participant A “I want to change, so I get back to the size that I was before, instead of all of this weight, a healthier weight, maybe get help staying away from fatty foods” Participant F	“[one participant] you made such a positive change in her life that now when we are discussing her future goals, one of her goals is that she says she wants to do a cooking program. And the reason she wants to do a cooking program is because she wants to learn how to eat healthy…So I just want to point out that the program has really made a positive change in someone’s life.” Carer 1

#### Programme problems and challenges

Participants with ID did not identify many areas for programme improvement, despite being explicitly asked. One participant stated finding that using the bike, “made me tired”, and another participant described struggling with motivation to get out of bed and attend the programme: “Maybe getting out of bed [to come, was hard]. I wanted to stay snuggly and warm and I didn’t want to get out of a warm bed” (participant F). Carer participants, however, identified several areas for programme improvement. These are summarised in Table 11.

**Table 11:** Programme problems and challenges: qualitative participant feedback

Programme challenge	Quotes from carer participants
*The programme was inadequately resourced:* While the costs of the programme sessions were covered by the research team, transport to and from the sessions as well re-imbursement for carer time, was not covered. These out-of-pocket costs created financial stress for the participating organisation	“There wasn’t any additional resources or, um, I don’t know, supports we had available to us for the program. Like it was, time that I had to put aside out of my week and the other carer had to do the same, and we had, you know, to stop other clients using the vehicle so that we could use the vehicle on a Thursday [to get to the program]. So it was a bit of a challenge because we had clients from all different parts of [the disability organization]…like on the days we couldn’t get a vehicle, the taxi to get us all there ended up being, like $120 bucks, just in a taxi to do a turn around there and back” Carer 2
*Communication between programme facilitators and formal and informal carers was inadequate*	“I did get a copy of handouts [from the program] because I would request it, but in other cases the guys were getting a copy of the handouts but I’m not sure if the support worker or the homes they were living were getting a copy as well. So you don’t know if the guys are taking the paper then they don’t want it anymore and then they trash it… the group homes or the families where they are living need to told, even if its on an email so the group homes or families have access to the same information because some guys are very particular about their things being touched.” Carer 1
*Issues with malfunctioning physical activity equipment created stress for some participants*	“Towards the end of the program the straps on the exercise bike on the seat broke, and, um, because they weren’t working for a few weeks, it just was a little bit difficult for some of our clients to say, use the exercise bikes without the straps because they’d got used to them.” Carer 2
*Healthy eating component of the programme was too theoretical*	“For the nutrition [sessions]….I think it would have been more beneficial for the guys to learn in practical ways about food… I think that the talking was good, but I just know from experience that they need practice…. so perhaps teaching them to make a healthy lunch would have been a bit better than to just talk about it… Because I know that they learn by seeing, by doing, by touching” Carer 1
*Lifestyle changes may not be sustained once programme is over:* While carers highlighted a number of benefits resulting from programme participation, they expressed concern that changes may be unsustainable without further buy-in from family members and paid support staff	“If they [participants with ID] don’t have a constant support, or a program in place with someone, or a group of people will be taking them every week to continue these [healthy lifestyle] approaches, …it just won’t happen. They won’t independently go and do it. Either they need the assistance to travel somewhere, or they need someone’s guidance to help them use the equipment in the gym, and …they need someone there to give them that push”. Carer 2 So I think that it’s got to be a real commitment, not just from the practitioners perspective but also from the families perspective, because without their support they can't really do it alone. The ones that did [make healthy lifestyle changes] had extra support, whether that was in a group home or it was at home. So, yeah, it’s got to be a group agreement, it’s not just the participant. Because if the participant wants to lose weight, they want to do exercise but they live in a group home unless the carer takes them out they wont be able to do exercise. Its compromising, it’s finding a comprise, within the organisations where they are living. And keeping them accountable as well, do you know what I mean?” Carer 1

## Discussion

Results from the *Get Healthy!* feasibility pilot indicate that the programme was well received by a small group of adult participants with moderate ID and their carers. The programme has potential to positively impact several indicators of cardiometabolic health.

### Reflections on programme feasibility

*Screening:* Only participants screened by GPs as safe to participate were included in the trial, however, GPs were not required to provide programme facilitators with details of each participant’s specific health conditions. Unfortunately, not all participants and/or carers in this feasibility trial were able to reliably self-report relevant medical conditions. For the planned efficacy trial we therefore recommend replacing the generic medical consent form, which only asks if any restrictions should be placed on the person’s participation, with a more detailed form prompting the GP’s to indicate whether or not the person has a known diagnosis of: high blood pressure, diabetes, asthma, allergies, cardiac complications, lipid abnormalities, musculoskeletal conditions, or psychiatric or behavioural issues that may impact on programme participation. GPs should also be requested to provide an up-to-date list of all medications the person is currently prescribed. Knowledge of these conditions can support programme facilitators to better manage risk and tailor the programme more effectively to each participant’s needs.

### Increasing programme engagement

While overall programme attendance rates were acceptable and mean engagement scores were high, participants were notably less engaged in the nutrition component of the programme, compared to the physical activity sessions. Qualitative feedback from the exit interviews suggests that decreasing didactic teaching content and increasing practical activities related to food choice and preparation may increase engagement in nutrition sessions for the efficacy trial. An additional issue detracting from programme feasibility was limited carer involvement. Only two carers regularly attended the programme with participants, and no clear channels of communication were established between programme facilitators and carers who did not attend. Prior research has highlighted that carer buy-in can significantly improve the extent to which people with ID engage in and sustain healthy lifestyle behaviours [37–39]. Developing supplementary on-line or other written teaching content that carers can engage with remotely and developing a schedule of home-visits by programme facilitators, may help to build closer relationships with carers during the efficacy trial.

#### Improving data collection

Problems arose with the completeness and/or reliability of data from several of the outcome measures used in the feasibility pilot. A number of factors are likely to have contributed to this issue: Firstly, several of the measures (i.e. 24-h food recall, food photography, accelerometers, IPAQ-pr) required considerable carer support to complete. Retrospectively it is clear that the pilot protocol did not include a sufficiently robust carer training and follow-up schedule to ensure that full data sets were collected. The carer handouts and instructions sheets, for example, were not necessarily passed on from the participants with ID to their home carers and *Get Healthy!* programme facilitators did not have access to home carer contact information.

Since food photography [40–42], use of accelerometers [43, 44] and the IPAQ-proxy [26] have all been shown in previous studies to be reliable and viable to implement in adult populations with ID, we recommend keeping these measures in the protocol for the efficacy trial. However, the protocol should be modified to allow programme facilitators to liaise directly with carers to provide them with task training. A schedule of telephone prompts and face-to-face support should also be implemented during data collection periods.

Secondly, it is possible that several of the trial outcome measures, specifically, NAKS, PWI-ID and 10RM strength testing, were too complex and therefore inappropriate for our study participants, whom had a more ‘moderate’ spectrum of ID. The planned 10RM physical strength testing, for example, was unable to be implemented due to cognitive difficulties participants experienced using even a simplified rate of perceived exertion scale. Despite our AEP using clinical judgement to determine endpoint of 10RM testing (e.g. facial grimacing, perceived exertion, and technique safety), we believe that the values obtained do not represent individual’s true 10RM. To increase trial efficacy, we recommend replacing this measure with an objective assessment with simplified protocol measures (and reduced risk), such as a hand-grip strength test for upper body strength. Functional testing parameters, inclusive of normative data validated within this population remains limited, with future research looking to widen appropriate assessment selection.

Similarly, the NAKS measure may have been too complex for several participants in this study. While the NAKS has been validated in populations with mild ID [35], it requires participants to be able to meaningfully choose from four options. We recommend that a pre-testing protocol be implemented in the efficacy trial to assess whether participants are capable of meaningfully choosing from four options. Another issue of concern that arose with administrations of the NAKS was presence of carers, who in some cases attempted to ‘prompt’ participants with correct answers. For the planned efficacy trial, we recommend administering the NAKS without a carer present wherever possible. Should the participant wish to have a carer present in a support capacity, we recommend providing additional guidance to the carer to refrain from prompting the participant’s answers.

In the PWI-ID validation study [45], which included adults with mild and moderate ID, all participants were able to be administered at least the most basic (2-point scale) index. However, in our pilot, baseline pre-testing identified participants who were unable to be administered even this 2-point scale. This finding suggests several of our participants may have had a greater degree of intellectual impairment compared to the validation study cohort. The other issue of concern we experienced with the PWI-ID involved participants passing the pre-testing phase but then scoring at the top of the response range across all seven domains. Such a scoring pattern is most likely the result of acquiescent responding, a known issue among populations with ID [46]. The original validation study for this measure [45] also encountered this issue with data from 32% of respondents needing to be removed prior to analysis due to suspected acquiescent responding. We recommend excluding suspected acquiescent response data from analysis in the efficacy trial. Participants who fail the baseline pre-testing protocol should not have the measure re-administered at endpoint.

### Reflections on potential programme efficacy

Efficacy data from the trial are exploratory in nature, given the small sample size, and multiple missing data-points. Preliminary findings, however, indicate that compared to baseline, most participants in the ‘Get Healthy!’ programme recorded clinically meaningful reductions in waist circumference and some improvement in measures of cardiovascular fitness. Some participants also displayed clinically meaningful improvements in physical strength at programme endpoint. BMI, quality of life, and objectively measured health literacy did not appear to improve from baseline to endpoint. Dietary intake patterns were mixed and analysis was limited due to incomplete data.

The decreases in waist circumference recorded for all but one participant is a promising finding, given that waist circumference provides a relatively simple and accurate reflection of central adiposity [47, 48]. Decreased central adiposity, in turn, is a strong predictor of lower risk for hypertension, diabetes mellitus, dyslipidemia, metabolic syndrome, and coronary heart disease [49, 50]. Reassuringly, given the lack of weight loss among study participants, this finding holds true irrespective of changes to BMI [50].

Study participants displayed some improvements in cardiovascular fitness from baseline to endpoint based on the YMCA sub-maximal testing protocol. Our participants significantly increased their clinical cardiovascular fitness throughout this intervention. Participants not only increased (178%) their duration of cycling (2.81 vs. 7.80 min) but also their workload (70.83 vs. 120 W) by 69% post-intervention, while maintaining a steady HR (70–65% APMHR). This indicates that participants were able to exercise longer at an increased workload, using the same amount of energy, indicating increased cardiovascular fitness. This is supported by the number of participants able to complete the YMCA sub-maximal testing protocol post-intervention (3 participants vs 0 participants pre-intervention). Based on post-intervention data, the average estimated VO^2^ was 2.14 L/min (31.51 ml/kg/min) indicating ‘poor’ cardiovascular fitness [51]. Poor cardiovascular ability to sustain prolonged physical work is a powerful predictor of morbidity and all-cause mortality as well as cardiovascular specific mortality [52, 53]. Improvements in measures of cardiovascular fitness, if confirmed in a sufficiently powered efficacy trial, would thus be another strong argument to implement the programme more widely among this at-risk population. Given that no participants were able to complete this incremental YMCA protocol pre-intervention, in addition to the poor cardiovascular fitness measured, we suggest fellow researchers consider the inclusion of a steady-state cardiovascular cycling protocol, such as the Astrand Rhyming Test, or modification to the YMCA step test to further increase data collection and efficacy.

Improvements in measures of physical strength were also noted for some participants from baseline to programme endpoint. Again, this result, if replicated in a sufficiently powered efficacy trial, would be promising in terms of cardiovascular risk reduction. Improved physical strength has been shown to have an attenuating effect on premature all-cause mortality [54], as well as lifestyle-related disease such as diabetes [55], stroke [56] and obesity [54]. Our physical strength data highlights poor upper and lower body strength for adults with ID. Of particular note, is lower limb endurance and falls risk, indicative through the 30-sec STS data. Despite our cohort having a mean age of 46, this 30-sec STS data indicates increased falls risk for adults aged 60–64 years. Despite our intervention showing clinically meaningful improvements (12.40 pre- vs. 15.33 post-intervention) in this outcome measure, post-intervention data continued to represent increased falls risk for an age bracket 14–18 years their senior, highlighting the need for continued exercise interventions and health supports in this population.

A point of further discussion includes the relatively large age range of the study participants (28–62 years of age). Despite concerted efforts of the research team to recruit people with ID 40 + years of age, due to the nature of the disability service providers who expressed interest in this study, we received a large age range of eligible participants. We must highlight the variances in physiological adaptations based on the ageing process, particularly on the ability to build muscular strength and improve cardiovascular fitness as a limitation of this study. This large age range could be a contributing factor in the diversity of change seen across our physical outcome measures. Further efficacy studies should look to either narrow the demographic age bracket of participants, or perhaps target the exercise intervention dependent on age.

## Conclusion

The ‘Get Healthy!’ feasibility pilot was well attended and positively received by participants and carers. Outcome data, while exploratory in nature, suggests the programme has potential to improve several important indicators of cardiometabolic health including waist circumference, cardiovascular fitness and physical strength. Problems with missing data-points and potentially unreliable data were identified, however, and several of the study outcome measures will require modification or replacement prior to implementing a full-scale efficacy trial. Further attention should also be given to improving carer buy-in to maximise data collection and programme impact and sustainability.

## Data Availability

The datasets used and/or analysed during the current study are available from the corresponding author on reasonable request.

## Abbreviations

AEP: Accredited Exercise Physiologist
BMI: Body mass index
CV Fitness: Cardiovascular fitness
ESSA: Exercise and Sports Science Australia
HEIFA: Healthy Eating Index for Australian Adults
ID: Intellectual disability
IPAQ-pr: International Physical Activity Questionnaire-proxy respondent
NAKS: Nutrition and Activity Knowledge Scale for Use with People with an Intellectual Disability
PWI-ID: Personal wellbeing index-intellectual disability
STS: Sit to stand
WC: Waist circumference
WFR: Weighted food record
